# Dataset of cattle biometrics through muzzle images

**DOI:** 10.1016/j.dib.2024.110125

**Published:** 2024-02-02

**Authors:** Syed Umaid Ahmed, Jaroslav Frnda, Muhammad Waqas, Muhammad Hassan Khan

**Affiliations:** a National University of Computer and Emerging Sciences FAST-NUCES, Karachi, Pakistan; b Department of Quantitative Methods and Economic Informatics, Faculty of Operation and Economics of Transport and Communications, University of Zilina, 01026 Zilina, Slovakia

**Keywords:** Livestock, Identification, Pattern recognition, Tagging, Farms

## Abstract

The Cattle Biometrics Dataset is the result of a rigorous process of data collecting, encompassing a wide range of cattle photographs obtained from publicly accessible cattle markets and farms. The dataset provided contains a comprehensive collection of more than 8,000 annotated samples derived from several cow breeds. This dataset represents a valuable asset for conducting research in the field of biometric recognition. The diversity of cattle in this context includes a range of ages, genders, breeds, and environmental conditions. Every photograph is taken from different quality cameras is thoroughly annotated, with special attention given to the muzzle of the cattle, which is considered an excellent biometric characteristic. In addition to its obvious practical benefits, this dataset possesses significant potential for extensive reuse. Within the domain of computer vision, it serves as a catalyst for algorithmic advancements, whereas in the agricultural sector, it augments practises related to cattle management. Machine learning aficionados highly value the use of machine learning for the construction and experimentation of models, especially in the context of transfer learning. Interdisciplinary collaboration is actively encouraged, facilitating the advancement of knowledge at the intersections of agriculture, computer science, and data science. The Cattle Biometrics Dataset represents a valuable resource that has the potential to stimulate significant advancements in various academic disciplines, fostering ground breaking research and innovation.

Specifications Table

**Table:** 

Subject	Animal Recognition, Livestock Identification, Computer Vision, Pattern Recognition, Data Science and Artificial Intelligence, Animal Biometrics
Specific subject area	Image based Biometrics, Identity Recognition, Tracking Records, Digital Identity, Biometrics Research, Animal Welfare, Animal Tracking
Data format	Raw: JPG and PNG (All data without pre-processing) Raw: JPG and PNG (Original)
Type of data	Images
Data collection	For the process of data collection, we used a HD Camera and two different mobile phones. The data collection process took about 6–7 months in early morning times **(5:30 am to 11:00 am) approximately. Cannon D80 (48 MP)** was used for High Quality Image capturing. While two mobile phone cameras namely **OnePlus 9 Pro** and **OPPO A76 (13 MP)** were used for capturing images as well. The process of manual annotation was conducted in order to assign labels to the photos using the Image software. The data was divided into two distinct groups, namely “dirty” and “muzzle”. The relevant information and comments were stored in text file formats, allowing for its application with various models. The dataset was partitioned into training, validation, and testing subsets that are appropriate for machine learning purposes. The dataset's size is 13.9 GB, and a ZIP file was provided to facilitate the downloading process.
Data source location	Institution: National University of Computer and Emerging Sciences FAST-NUCES City: Karachi Country: Pakistan Latitude and Longitude for collected samples: 24° 53.625311′ N and 67° 11.707523′ E, Elevation: 104 m/341 feet
Data accessibility	Repository name: Cows Frontal Face Dataset Data identification number: 10.5281/zenodo.8377921 Direct URL to data: https://zenodo.org/records/10535934

## 1 Value of the Data

• The utilization of muzzle data in cattle offers a distinctive and immutable biometric identification, comparable to the concept of fingerprints in humans. The distinctiveness of this characteristic holds significant value in the context of tracking and identifying individual cattle, hence facilitating precise record-keeping throughout expansive herds.
• Alterations in the muzzle of cattle can serve as indicators of health conditions. Through the examination of muzzle data over a period of time, professionals in the field of veterinary medicine and agriculture are able to engage in proactive monitoring practices, hence enabling the detection of indications pertaining to diseases or infections. This approach facilitates early intervention strategies and subsequently enhances the overall health of livestock populations.
• The integration of muzzle data into livestock management systems enables the automation of various processes, like as feeding and medicine distribution, within the context of livestock management. The implementation of this approach significantly improves operational efficiency and mitigates the likelihood of errors in extensive cattle farming endeavors.
• Traceability and quality assurance are crucial aspects in the meat and dairy industries, as they play a significant role in ensuring consumer safety and maintaining product quality. Muzzle data possesses the capability to facilitate the tracing of product origins, hence assuring compliance with safety and quality standards.
• The utilization of muzzle data in genetic studies enables researchers to discern features that are correlated with particular cattle breeds or health issues. This information can provide valuable insights for breeding programs aimed at enhancing the health and productivity of cattle.

## 2 Data Description

The dataset includes different cattle (mix breeds) images collected in early morning times in a total of 6–7 months. Three different cameras are used for the collection of dataset. The different cameras are used for having the variance in image quality. All the different resolutions and camera specifications are reported in Table 1.

**Table 1:** Camera and mobile devices specifications used for image capturing.

Device	Name	Specifications	Resolutions
Camera DSLR	Canon D800	4 K/60fps Video shot and 48MP camera	3648 × 5472
Mobile Phone 1	One Plus 9 Pro	48MP, Laser AF	3008 × 4000
Mobile Phone 2	Oppo A76	13MP, FOV 80°, 5P lens, and AF	2336 × 4160 and 2608 × 4624

While capturing the dirty images of muzzles are also kept in the dataset in order to provide the application of noise removal in future. The dirty muzzles (noses) are present in some of classes with the complete face for future research to explore the content with full potential.

## 3 Experimental Design, Materials and Methods

**Fig. 1:**
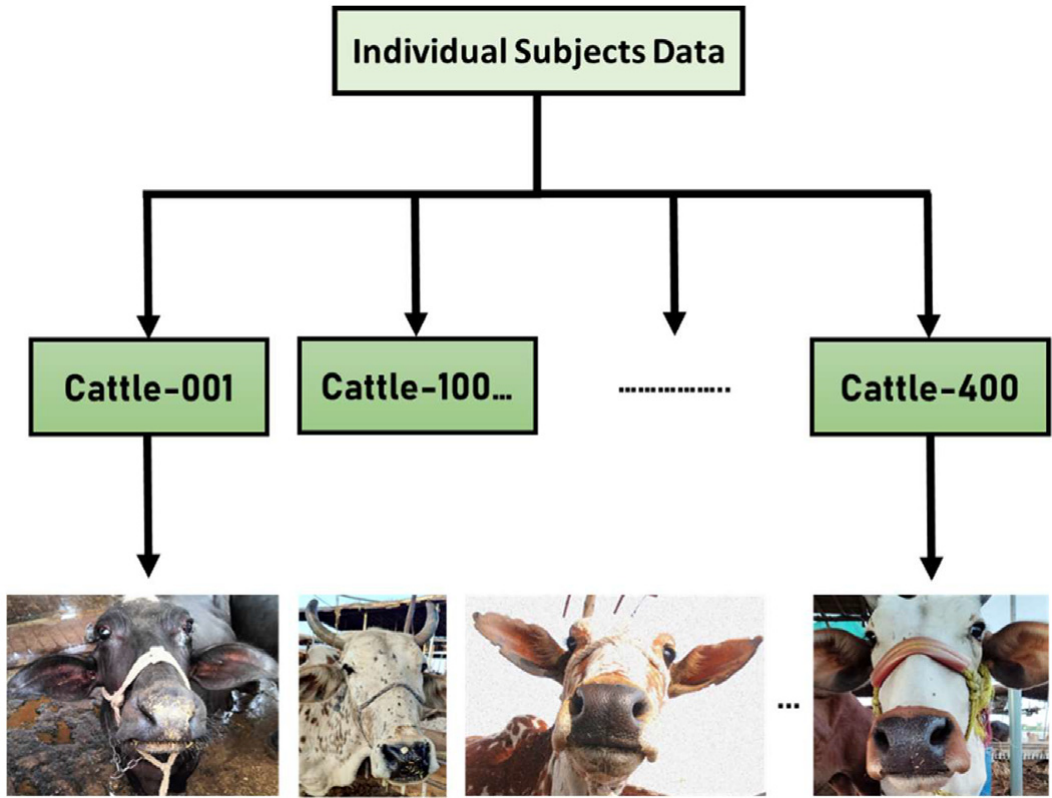
Folder structure of cattle biometrics dataset.

Animal identification is a critical task in various domains, including agriculture, veterinary sciences, and wildlife conservation. Traditional identification methods, such as paint [1], ear tags [2], microchips, ironing, freeze branding and tattoos, have limitations in terms of practicality, accuracy, and scalability and cause distress in cattle [3]. Using microchip requires injection [4] or injection of any other kind of sensor within body [5] with which it can be located. Over the past few years, the development of deep learning methods and advancements in computer vision have created opportunities for advancements in individual animal identification. In order to achieve precise recognition of cattle faces, it is imperative to employ face detection techniques [7]. Muzzle patterns have biometric details like fingerprints in humans which can be highly accurate for identification [6,8], but image of animal should be taken from close so that muzzle patterns must be clear. A significant difficulty faced by biometric algorithms is meeting the demanding accuracy standards, especially when dealing with extensive and varied populations of cattle breeds. An innovative approach for animal identification focuses on utilizing muzzle images as a unique biometric marker for individual beef cattle identification. Convolutional neural networks (CNNs), a type of deep learning technique, have been deployed, to extract discriminative features from muzzle images [10]. By conducting experiments, this approach has proven its efficacy by achieving a notable degree of accuracy in identifying individual cattle based on muzzle images. This result offers significant insights into the possibilities and potential benefits of employing computer vision techniques for identification of cattle. A vision-based hybrid approach for animal face identification uses a technique that enables knowledge transfer from pre-trained models, to develop accurate and robust animal face recognition models. By combining deep learning techniques with traditional computer vision methods such as feature extraction and image alignment, the hybrid approach seeks to improve the performance and generalization capabilities of the animal face identification system. [9]. The method emphasizes the benefits of combining deep learning methods with computer vision techniques for animal identification. By utilizing muzzle images and animal face identification, we can improve the precision and dependability of individual animal identification systems. The incorporation of deep learning techniques, specifically convolutional neural networks (CNNs), enables the extraction of significant features from muzzle images, enabling accurate identification of individual cattle. [10]. Our work and dataset is limited to Muzzle Detection. The recognition part will be exploring in future using the noise removal and deep learning techniques in the next versions utilizing the same dataset (Fig. 1).

A limited number of data sets are accessible for analysing cow muzzle, with only two publicly available datasets [9,11] from Australia and United States. Additionally, there is no existing method for identifying cows using AI and computer vision, which means that there are no real-time photos available for training a model. The majority of the dataset used in our research has been collected by our team. We have collected the **largest dataset of cattle** in number of subjects in Pakistan.

### 3.1 Dataset collection

We have made available a dataset of **2893** images taken from **459 distinct cows**, which we believe to be the largest and most extensive publicly accessible biometrics dataset for livestock. We have produced dataset consisting of about 459 classes of cattle.

### 3.2 Image pre-processing

Once the photos were collected, we prepared them for the cow recognition model. This involved a process called image pre-processing, which comes after capturing the images. In order to ensure the efficient operation of computer vision and deep learning models, it is crucial to pre-process the images. The dataset is increase to 8000 images after image augmentation. Image pre-processing helps us enhance the data and make pixel-level adjustments. It ensures model robustness, adaptability and makes sure that it doesn't over-fit the data.

### 3.3 Image annotation

After collecting the cow photos, we prepared them for our cow recognition model. We made adjustments to the images to ensure the model works well. For this annotation, we used a user-friendly tool called LabelImg. LabelImg is a GUI tool written in Python and is designed for annotating images for object detection models. We manually annotated bounding boxes around the cow muzzle and labelled each box as muzzle. In this way, we teach the model to recognize different conditions of cow muzzles. The annotated files were saved in the YOLO format.

### 3.4 Image augmentation

Next, we proceeded to use a platform called Roboflow. Roboflow is a tool designed for assistance in annotation and augmentation of datasets, making it suitable for training models and is most commonly used in Deep Learning and Computer Vision tasks. Additionally, it offers options for training the model as well but we only used this for image augmentation.

### 3.5 Model training

In our research, we utilized the YOLO (You Only Look Once) algorithm, which is a cutting-edge object detection framework in computer vision. YOLO is highly notable for its real-time and efficient performance, achieved by utilizing a single neural network to simultaneously predict object bounding boxes and their corresponding. The parameters of YOLOv7 model are shared in Table 2.

**Table 2:** Parameters of the object detection/nose ROI selection model.

Hyperparameter	Value
Model	YOLOv7
Train Set	939
Test Set	140
Validation Set	82
Learning Rate	0.01
Momentum	0.937
Epochs	120
Optimizer	SGD

### 3.6 Muzzle detection results

Major outcome of YOLOv7 object detection model was that whether there is a muzzle or not in captured image. In order to test the model, we selected a random image and ran the model on that to check the results. The final values of results are given in Table 3.

**Table 3:** Outcomes and final training results of YOLOv7 model.

Parameters	Value
mAP@0.5	0.995
mAP@0.95	0.759
Training Time	2.45 H
Precision/Recall	0.99

## Limitations

None.

## Ethics Statement

All animal data used in this study were collected as part of standard farming and selling practices. As such, no part of this research was subject to approval of an ethics committee.

## CRediT authorship contribution statement

**Syed Umaid Ahmed:** Conceptualization, Methodology, Software, Writing – original draft, Investigation. **Jaroslav Frnda:** Validation, Writing – review & editing. **Muhammad Waqas:** Validation, Writing – review & editing. **Muhammad Hassan Khan:** Supervision, Validation, Writing – review & editing.

## Data Availability

Cows Frontal Face Dataset (Original data) (Zenodo).
